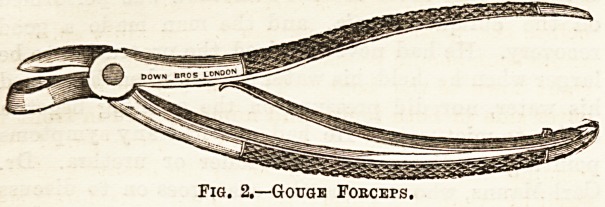# Acute Necrosis

**Published:** 1896-02-29

**Authors:** E. Percy Paton

**Affiliations:** Hon. Surg. to the Mildmay Hospital, Bethnal Green.


### Medical Progress and Hospital Clinics.

[*The Editor will be glad to receive offers of co-operation and contributions from members of the profession. All letters should be addressed to* The Editor, at the Office, 428, Strand, London, W.C.]

### ACUTE NECROSIS.

By E. Percy Paton, M.D., M.S., F.R.C.S., Hon. Surg. to the Mildmay Hospital, Bethnal Green.

In order to obtain a clear understanding of the clinical features of acute necrosis (otherwise known as acute infective periostitis, or osteomyelitis, &c.), it is necessary first to consider what is the now generally-accepted view as to its pathology. And here it may be stated at once that the part of the bone affected is the active, cellular, bone-forming tissue which forms the deep layer of the periosteum, fills the Haversian spaces, and is also present in the marrow of the bone and forms the actively growing part of its structure. Since this cellular tissue is most active during adolescence, it would be natural to expect that its liability to become diseased would be greatest during the period of life before puberty, and this is what is found in actual practice, acute necrosis being rare in adult life. The course of events may be described typically as follows: In a patient, most commonly a boy below the age of puberty, whose general condition is already below par, it may be from a recent attack of measles or whooping cough, or from some other cause, some bone—such as the femur or tibia—receives a slight injury, by which the vitality of its active cellular tissue is lowered, and at this spot micro-organisms which have, in his already debilitated condition, found an entrance into his circulation, probably by the lungs or alimentary tract, and which are capable of giving rise to pus formation, find a suitable nidus to grow in, either beneath the periosteum (acute periostitis) or in the medulla of the bone (acute osteomyelitis), where they rapidly set up acute suppurative mischief. The subsequent results will depend on the situation of the trouble, the virulence of the organisms, and the resistance of the patient's tissues.

In a typical case the symptoms of the disease are the following: A child—most commonly a boy, possibly from their increased exposure—receives a slight injury, which is most probably so slight that it is forgotten almost as soon as received. A day or so afterwards the patient is suddenly attacked with an acute illness, like the onset of a specific fever. There is high temperature, not infrequently a rigor or convulsion, with often vomiting or diarrhœa, headache, &c. At first no local symptoms are discoverable; but at the end of about thirty-six or forty-eight hours, or it may be a little longer, acute tenderness, swelling, and œdema are noticed over one of the long bones, especially towards one or other extremity. In a few days this rapidly increases, so that at the end of a week, if the case be left to itself, fluctuation is discoverable, pus having formed between the periosteum and the bone. This pus rapidly spreads upwards and downwards along the shaft, stripping up the periosteum until it reaches the epiphysial cartilage, to which the periosteum is attached, when it burrows in between the epiphysis and diaphysis, separating them from each other. Should this occur, soft crepitus will be felt on movement of the limb; while, should the whole shaft be affected and both articular ends separated, the shaft will be left loose in a bag of pus. Before, however, this occurs, the periosteum usually gives way at some spot, and the pus burrows and spreads in the cellular planes of the limb and a large abscess is rapidly formed, which, if the case be still left to nature, may ultimately discharge itself through the skin in one or several places—or, as is much more frequent, is let out by incisions. During all this time the temperature remains high and the general condition of the patient grave. So soon, however, as the pus is allowed to escape, if free drainage be established and there be no complications, the temperature falls to normal, or almost so, and with this the local swelling and pain, as well as the constitutional disturbance, subside, and what is now left is one or more openings, discharging at first considerable quantities of pus, and leading down to bare bone. What will happen to this bone is at first doubtful; as one of the names of the disease implies, necrosis is a very constant, though not an absolutely certain result; especially may this be averted if the medulla be but little if at all affected, and if the treatment be active and efficient. Should necrosis occur, only time will show what extent of bone will be involved, and this may vary from a small scale to practically the whole shaft of a long bone. The subsequent course of the case is extremely chronic; while the dead bone is being separated from the living all the active inflammatory symptoms disappear, and there are left discharging sinuses leading to the dead portion, around which is deposited from the periosteum a layer of spongy bone which ensheathes and closes it in, except in one or two places, where openings known as cloacæ, through which the discharges escape, are left. This goes on until the dead bone becomes loose and is removed, when the ensheathing bone consolidates to take its place, and the openings close up. Often, however, the closure in the first instance is only temporary, not all the dead part having been removed at once, and the sinus reopens again until this is effected, and the sinus finally closes, which satisfactory result may not ensue until very many months, or even longer, have elapsed from the original onset of the disease.

Fig. 1.—Diagram of necrosed humerus enclosed in new bone laid down from periosteum with cloacæ.
a, Periosteal new-bone; b, dead shaft; c, cloacæ.

The *complications* of this disease add very greatly to its gravity. A possibility always to be borne in mind is that of there being more than one bone affected at the onset, and so a careful investigation should be made of all the long bones. The other complications can often be averted by early active treatment. The ones most to be feared are septicæmia and pyæmia, which manifest themselves, the former in persistent high temperature, with grave general symptoms, sometimes with a petechial rash, the sufferer rapidly falling into a typhoid condition, the latter in local suppurations coming on with rigors. With pyæmia also in these cases there is a special liability to get inflammations usually suppurative of the pleura and pericardium. These arise most probably from the bursting of small miliary abscesses which have formed on the surface of the lungs or heart. If one considers the relations of the veins to the inflamed bone in cases of acute periostitis, it is not difficult to see how, due to their passage through the compact tissue, they will be unable to collapse and so empty themselves, and should inflammation spread to them clotting will take place, which clots will rapidly become infected with micro-organisms from the infected bone, and these septic clots spreading to the neighbouring veins, portions will be carried away into the general circulation, giving rise to the troubles above described, wherever they may lodge, by giving rise to small infarcts, which are from their formation infected with the micro-organisms which cause suppuration. Another local complication is acute arthritis, caused by the pus finding its way into the joint at one or other end of the affected long bone. This it may do either by perforating the epiphysis or by making its way into the joint through the soft parts. The immediate result is acute pain on any, even the slightest, movement of the joint, accompanied by rapid infiltration of all the structures surrounding it, as well as those entering into its immediate composition, resulting in most cases in a rapid and complete disorganisation of the articulation, with at the same time very severe general symptoms. This, however, is not always the case, as there is a variety of these cases, occurring especially in very young children, in which the joint may be affected, and yet, if it be freely drained, its functions are not destroyed. The original disease here is usually confined to the epiphysis, and is frequently unrecognised until the joint is already involved, and is often called the acute arthritis of infants.

**Diagnosis.**—It is very important that the disease should be recognised early, as prompt treatment has such a very marked effect on its course. At its very commencement, before the local symptoms have had time clearly to develop, it is most likely to be taken for one of the specific fevers or acute rheumatism. The appearance of marked local symptoms will clear up the doubt as to whether one of the exanthemata is coming on, and the fact that the swelling, pain, and local inflammatory symptoms are not primarily connected with a joint will show that acute rheumatism is not present. In some cases the most noticeable of local symptoms, especially in small children, is the fact that the patient does not move one of its limbs at all, and it is only by careful examination that the cause of this pseudo-paralysis is discovered in a swollen, tender, inflamed bone, this discovery being still further hampered by the frequent presence of a considerable amount of subcutaneous adipose tissue in these small children. The only other trouble for which acute periostitis is likely to be mistaken is cellulitis, but as an incision will be required in both cases the treatment will speedily clear up the diagnosis.

**Treatment.**—That this should be undertaken early and should be active is most important. As soon as the nature of the case is clearly made out, and even before the presence of pus can be certainly affirmed, an incision should be made into the swelling extending right down to the bone. This is best made on the outer side in both femur and humerus, and in front if the tibia is affected; but the exact situation of the opening will be more influenced by the situation of the swelling than by purely anatomical considerations, except in so far as to avoid the large vessels and nerves. Should no pus be found great good will still have been done, as the relief of tension may prevent its formation; while, if it has already been formed, providing for it a free exit will prevent its stripping up the periosteum over a large area of bone, and so limit the mischief as far as may be, and remove the probability of a neighbouring joint becoming affected. Should it be necessary for free drainage, counter openings must be made and tubes put through, so that the cavity may be frequently and easily irrigated. In many cases this treatment will cause the temperature at once to fall and the general condition to improve. Sometimes, however, this is not the case, and the cause is then to be sought in one of the following conditions: The commonest is imperfect drainage of the cavity, and this is easily remedied by making further openings and instituting freer irrigation. Two other local causes are, infection of a joint, and the fact that in a particular case the medulla of the bone has been the part on which most of the stress of the disease has fallen, and the medullary cavity, of course, is not drained by mere incision. If a joint be affected free drainage must at first be tried, but if the patient still goes down hill recourse will be necessary to amputation, which should then, if possible, be done so as to remove the whole of the affected portion of bone as well as the disorganised joint. If osteomyelitis be the cause of the high fever, it is best, either with a large trephine or other means, to make an opening into the medullary cavity, so as to provide drainage and relieve tension; or in some cases it may be necessary to remove the greater part of the diseased diaphysis, or even the whole of it, should the disease have so extended as to isolate it from the articular ends of the bone. Removal of the diaphysis should, if possible, however, be avoided for two reasons; firstly, that in most cases more of the shaft will be removed by doing so than would ultimately die and require removal, as the amount cannot at this stage be estimated with certainty, and secondly, if the old bone, even though it should die, be removed too early, there will be no scaffolding left round which the new bone formed from the periosteum can be modelled.

Another cause which may give rise to the temperature keeping up is a general septic infection, which may occur either as a septicæmia or with localised suppurations as pyæmia. These very grave complications must be treated as general principles, a supporting line of treatment being adopted, and any localised collections of pus opened as they occur.

After all the acute inflammatory symptoms have passed off there are left one or more sinuses leading down to bone which is bare, of which a larger or smaller portion in almost all cases, except those of the slightest severity, dies. This dead portion is gradually separated by ulceration of the living bone with which it

Fig. 2.—Gouge Forceps.

is in contact, and as this is naturally a slow process extending, if the necrosed portion be of any size, over four or five or more months, all that can be done until the sequestrum is loose is to keep the sinuses cleaned and dressed, which is easily done, as the amount of discharge is usually small. To obtain an idea as to whether the sequestrum is loose or not, an attempt may be made to move it by judiciously-applied pressure with a probe-pointed director, or with two such, pressing alternately on the bare bone through different sinuses. When this is found to be the case, or when a sufficient time has been judged to have elapsed, one or more of the sinuses is slit up, and the cloaca through the ensheathing bone enlarged with ordinary bone forceps or gouge forceps (see fig. 2), the loose sequestrum is then seized with sequestrum forceps and removed. If it be of very large size it may sometimes be better to divide it into two pieces rather than sacrifice too much of the new bone, on which the integrity and usefulness of the limb will so much depend, by cutting a large opening to admit of the dead piece being removed intact. When all the dead bone has been removed the new bone soon consolidates and forms often a very good substitute for the old one, while the sinuses close up.

## Figures and Tables

**Fig. 1. f1:**
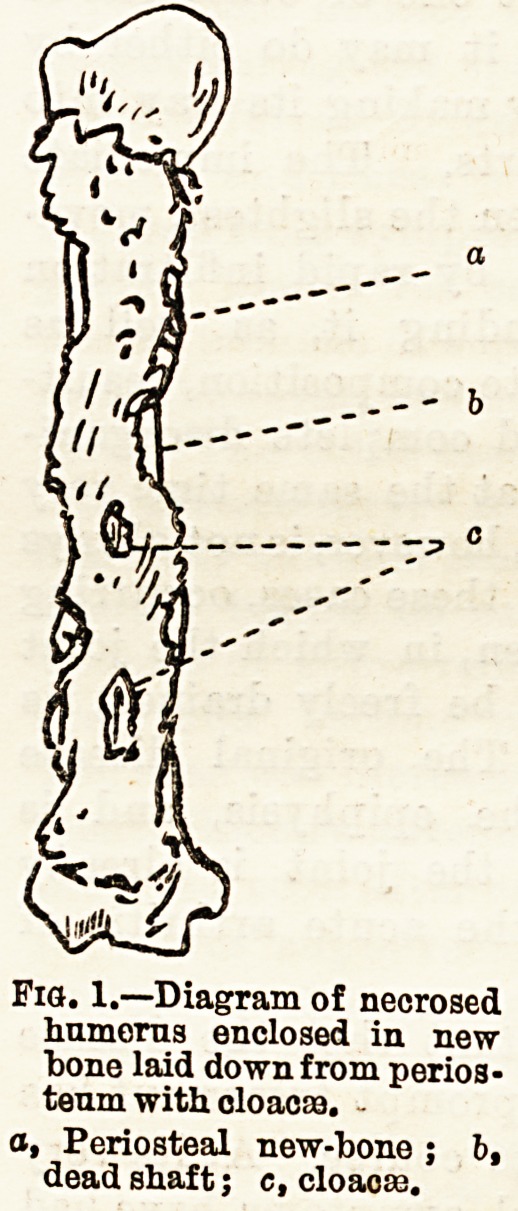


**Fig. 2. f2:**